# Calcitonin gene-related peptide (CGRP)-induced recovery after myocardial infarction: Is there a role for CGRP-targeted molecular image-guided strategies in cardiology?

**DOI:** 10.1007/s12350-021-02686-8

**Published:** 2021-06-04

**Authors:** Rudolf A. Werner, Takahiro Higuchi

**Affiliations:** 1 Department of Nuclear Medicine, University Hospital Würzburg, Oberdürrbacherstr. 6, 97080 Würzburg, Germany; 2 Comprehensive Heart Failure Center, University Hospital Würzburg, Würzburg, Germany; 3 OkayamaUniversity Graduate School of Medicine, Dentistry and Pharmaceutical Sciences, Okayama, Japan

As a potential vasodilator,^1^,^2^ the discovery of the Alpha-Calcitonin Gene-Related Peptide (CGRP) immediately triggered studies to investigate potential cardioprotective effects, e.g., in dilated cardiomyopathy.^3^ Therefore, in the present study, *Bentsen* et al aimed to investigate the effect on cardiac recovery after administration of a systemically applied alpha-CGRP analogue (SAX) in rats after myocardial infarction (MI).^4^ Relative to placebo-treated controls, the SAX group had improved cardiac defect sizes during long-term follow-up, as assessed by 99mTc-sestamibi SPECT.^4^ Moreover, in rats that died in the subacute phase early after MI, the SAX group demonstrated a significant longer survival time relative to placebo.^4^ The authors concluded that in rats, SAX has a cardio-beneficial effect after permanent coronary occlusion.^4^ The present study provides not only insights in SAX-related beneficial long-term outcome in animals after MI but also on prolonged survival in a subacute phase early after the acute event. In this regard, the authors extended the current knowledge on CGRP treatment in the context of myocardial ischemia in vivo.^4^,^5^*Källner* was among the first to prove that exogenously administered CGRP can improve post-ischemic coronary flow early after MI, but the authors could not provide evidence on a cardioprotective effect of CGRP during long-term follow-up.^5^ The authors of the present study, however, used SPECT as an endpoint to assess infarct size three weeks after the acute event, demonstrating smaller perfusion defects, thereby suggesting that SAX can also improve long-term cardiac outcome.^4^ However, to further provide more evidence whether SAX treatment would be indeed useful to improve cardiac performance in a chronic phase post-MI, future studies should use combined multimode endpoints to provide more functional information, e.g., by also implementing cardiac magnetic resonance (MR, including ventricular volumes or ejection fraction) 6 or using PET perfusion radiotracers, such as ^18^F-flurpiridaz providing more details on the extent of local ischemia.^7^,^8^

Bentsen et al decided for a permanent coronary occlusion model, as this may lead to an increased defect size and severity of local ischemia in the present proof-of-concept study.^4^,^9^ Future studies, however, may also investigate a dedicated coronary occlusion-reperfusion model closely mimicking the setting of percutaneous coronary intervention in patients, which may be also more clinically relevant.^9^ Such future studies may then also correlate the infarct size (e.g., assessed by SPECT) to immunohistochemistry, e.g., using triphenyl tetrazolium chloride or by assessing associations with CGRP plasma levels.^5^

Moreover, beyond using SPECT for the assessment of infarct size as a potential endpoint, it would be also interesting to use the recently introduced CGRP-targeted PET radiotracer 11C-MK-4232 in the present study setting.^10^ For instance, 11C-MK-4232 could be applied to SAX-treated animals vs placebo.^4^,^10^ Such an approach would allow to assess the retention capacities of SAX or other CGRP / vasodilating analogues prior to treatment on-set.^11^ For instance, a CGRP-related treatment could then be initiated at an increased 11C-MK-4232 radiotracer signal and compared to off-peak-treated animals post-MI, i.e., when the image biomarker signal has been dissipated.^12^ Such an elaborated approach of a molecular image-guided treatment strategy has been recently evaluated by targeting the C-X-C motif chemokine receptor 4 using the PET image agent 68Ga-Pentixafor in mice after MI, demonstrating that CXCR4 inhibition at the maximum of the target expression has a beneficial outcome when compared to off-peak-treated animals.^13^ To date, it remains unclear whether such an image-guided strategy may also work for targeting CGRP using PET technology. First in-human studies, however, reported on a high safety profile of 11C-MK-4232, and these encouraging results may trigger future studies in patients post-MI using this CGRP-targeting image agent, preferably in a translational set-up.^14^ In this regard, the short half-life of C11 (20 min) would make a clinical use rather challenging, and therefore, F18-labeled CGRP-directed radiotracers (half-life, 110 min) would be needed.^15^ Nonetheless, the present work of *Bentsen* et al lays the groundwork for future studies investigating the concept of image-guided treatment strategies post-MI, e.g., in a preclinical model after transient coronary occlusion randomizing animals to an on-peak group (treated at the maximum of the 11C-MK-4232 signal in the infarct) vs an off-peak group.^13^ Moreover, CGRP has been also advocated to play a potential role in cardiorenal interactions. For instance, it has been shown that deletion of the CGRP gene causes hypertension-induced end organ damage in both the heart and kidneys.^16^ As such, after primary cardiac damage, potential benefits for renal endpoints after CGRP treatment could be also assessed,^12^,^17^ e.g., by implementing additional kidney MR to investigate improvement in renal perfusion due to SAX-caused vasodilatation in the kidneys.^18^
